# Clinical Lectures on Surgery

**Published:** 1856-01

**Authors:** 


*Clinical Lectures on Surgery.* By M. Nelaton. From notes taken by W. F. Atlee, M. D. Phila—published by J. B. Lippencott & Co.

Dr. Atlee, whilst in Paris, attended upon the surgical lectures of Nelaton, who is one of the most brilliant Surgeons of France, and differs from many others of his countrymen, in the absence of all recklessness, which feature is a too prominent one among the Surgeons of that country.

There is in this work, an evident effort on the part of Dr. Atlee, to give a literal copy of these lectures, which renders them more valuable, because we are assured of their correctness. It would appear that these notes were taken at the Clinical Hospital, and doubtless translated before he lost the spirit of the lecturer's remarks. This required much labor, and the result is that we have a valuable work, and one which will be perused with profit.

We would advise all to procure this work, and particularly those who have a *penchant* for the use of the scalpel; and if all those who leave school, fresh from their Alma Mater, and intend to devote their time and attention to the art of Surgery, will but procure a copy, the sale will be extensive, for nearly every *tyro* dreams of no glory so bright and dazzling as that which is secured in the department of surgery.

We have often and again smiled at this piece of mistaken vanity and ambition, for there is infinitely more skill required at the bed-side of the sick, in correctly diagnosing some occult and complicated case, than in cutting into tissues, the numeral acquaintance with which is acquired, as we acquire a knowledge of the geography of the country, for the extirpation and removal of some malignant tumor, or even the performance of some of the capital operations. The world is inclined to give great eclat to the Surgeon, while on the other hand, some of the diseases to which "flesh is heir," is infinitely more difficult to control and manage, requiring more thought, judgment, skill, acquirement, and science, than any feat of the Surgeon. Into this error young beginners fall.

But as this is a digression, we would advise all to put themselves in possession of this work, and if so, they will feel toward Dr. Atlee a debt of gratitude.

